# Emotions on the loose: emotional contagion and the role of oxytocin in pigs

**DOI:** 10.1007/s10071-014-0820-6

**Published:** 2014-11-11

**Authors:** Inonge Reimert, J. Elizabeth Bolhuis, Bas Kemp, T. Bas Rodenburg

**Affiliations:** 1 Department of Animal Sciences, Adaptation Physiology Group, Wageningen University, P.O. Box 338, 6700 AH Wageningen, The Netherlands; 2 Department of Animal Sciences, Behavioural Ecology Group, Wageningen University, P.O. Box 338, 6700 AH Wageningen, The Netherlands

**Keywords:** Behavior, Emotions, Emotional contagion, Empathy, Oxytocin, Pigs

## Abstract

We studied emotional contagion, a simple form of empathy, and the role of oxytocin herein in pigs. Two training pigs per pen (*n* = 16 pens) were subjected to a positive treatment (pairwise access to a large compartment filled with peat, straw and some chocolate raisins) and a negative treatment (social isolation in a small compartment) in a test room using a within-subjects design. Thereafter, two naive pen mates joined the training pigs in the test room, but were not given access to the treatments. This allowed testing for emotional contagion. Subsequently, the naive pigs, serving as their own controls, were given 24 IU of oxytocin or a placebo intranasally 30 min before accompanying the training pigs, which were exposed to either the negative or positive treatment, to the test room. Behavioral differences found between the positive and negative treatments (e.g., play and “tail wagging” vs. standing alert, urinating, defecating and ears backward) show that the treatments induced a positive and negative emotional state in the training pigs, respectively. Changes in behaviors of the training pigs with and without naive pigs present (e.g., in ears backwards) and of the naive pigs with and without training pigs present (e.g., in standing alert) indicated that emotional contagion occurred, especially during the negative treatment. Oxytocin did not seem to affect the behavior of the treated naive pigs, but did affect behaviors (e.g., defecating) of the training pigs which had not received oxytocin. This suggests a role for oxytocin in pig communication, which merits further research.

**Electronic supplementary material:**

The online version of this article (doi:10.1007/s10071-014-0820-6) contains supplementary material, which is available to authorized users.

## Introduction

Empathy is recognized as a multilayered phenomenon (de Waal 2008; Preston and de Waal 2002) which can be defined as “the capacity to be affected by and share the emotional state of another, assess the reasons for the other’s state and identify with the other, adopting his or her perspective” (de Waal 2008). At the most simple level of empathy, emotional contagion, only the emotional state of the other is shared, but no cognitive perspective takes place (de Waal 2008; Preston and de Waal 2002). Emotional contagion is perhaps best illustrated by the situation in which the cry of an infant induces other infants to start crying too (Geangu et al. 2010; Simner 1971), because it shows that the other infants share the distress of the first infant, but they do not understand why the first infant started to cry. Sharing another’s emotional state is thought to be essential for group bonding and communication (Spoor and Kelly 2004; Špinka 2012). For instance, present danger may be noticed by one member of the group, and via emotional contagion, the other group members are alerted, thereby increasing survival chances of the whole group. Moreover, as emotions may serve to direct individuals to perform a specific task, sharing each other’s emotional state may facilitate coordination between individuals within a group. Emotional contagion is considered to be the phylogenetically oldest level of empathy (de Waal 2008; Preston and de Waal 2002). Hence, it is likely that emotional contagion is not a process confined to humans, but exists in many different animal species (de Waal 2008; Špinka 2012). Indeed, emotional contagion has been described to occur in, for instance, dogs (Custance and Mayer 2012), primates, birds, rats and mice (reviewed in de Waal 2008; Edgar et al. 2012a; Panksepp and Lahvis 2011).

The peptide oxytocin is traditionally implicated in parturition and lactation (Uvnäs-Moberg 1998). At present, however, it is also known that oxytocin plays a role in various social processes such as bond formation, social support and trust (Bartz and Hollander 2006; Lim and Young 2006). Moreover, oxytocin is suggested to play a role in processing emotional information (Graustella and MacLeod 2012) and in emotional contagion (De Dreu 2012; Shamay-Tsoory 2011). For instance, Hurlemann et al. (2010) found that human male subjects that were given an intranasal administration of oxytocin were emotionally more affected by photographs of other humans expressing a range of emotions, positive and negative, than subjects that received a placebo. That oxytocin could play a role in emotional contagion is very plausible, because oxytocin has been shown to exert effects on brain regions such as the amygdala, anterior insula, anterior cingulate cortex, inferior frontal gyrus and inferior parietal lobe (De Dreu 2012; Sofroniew 1983; Zink and Meyer-Lindenberg 2012), all of which seem to be involved in emotional contagion in humans (Bastiaansen et al. 2009; Preston and de Waal 2002; Shamay-Tsoory 2011; Singer 2006).

Pigs and other farm animals in intensive husbandry systems are usually kept at high stocking densities in a confined space (Spoolder et al. 2000; van de Weerd and Day 2009). Moreover, they are also commonly subjected to standard management procedures such as mutilations (e.g., tail docking), abrupt weaning, regrouping and transport from which they cannot escape and which lead to distress (e.g., Dudink et al. 2006; Geverink et al. 1998; Noonan et al. 1994; Stookey and Gonyou 1994). Under such housing conditions and management procedures, the chance of being affected by the distress of their group members is therefore relatively high. Apart from being affected by the distress of their group members, farm animals may also be affected by positive emotional states of their group members during, for instance, times of play (Held and Špinka 2011; Špinka 2012). The extent to which they are affected depends, however, on their capacity for empathy or emotional contagion (Edgar et al. 2011). Emotional contagion has to the authors’ knowledge only very sparsely been studied in farm animals [sheep (Anil et al. 1996; Colditz et al. 2012; Edgar et al. 2012a), chickens (Edgar et al. 2011, 2012b) or, more specifically, in pigs (Anil et al. 1997; Düpjan et al. 2011; Reimert et al. 2013)]. Both Anil et al. (1997) and Düpjan et al. (2011) found no evidence for emotional contagion in pigs, but that could have been due to their experimental design. In addition, they have studied emotional contagion of negative emotional states only. In Reimert et al. (2013), a different design was used to study emotional contagion of negative as well as positive emotional states during anticipation and during positive and negative treatments. With this design, some evidence of emotional contagion was found, but results were still rather subtle.

The first aim of the present study was, therefore, to investigate whether pigs show the capacity for emotional contagion. To that aim, the same experimental design as in Reimert et al. (2013) was used, but with some modifications that we expected to result in a clearer differentiation in behaviors during negative and positive anticipation and treatment, such as a prolonged training period. The second aim was to investigate whether oxytocin could play a role in emotional contagion in pigs. Based on our previous study (Reimert et al. 2013), we hypothesized that emotional contagion does indeed occur in pigs (i.e., that the emotional state of pigs as reflected in their behavior would be affected by the emotional state of their group members) and, based on the literature, that oxytocin makes emotional contagion stronger both in a positive and negative way (De Dreu 2012; Hurlemann et al. 2010).

## Methods

### Subjects and housing

For this study, 96 Pietrain × (Great Yorkshire × Dutch Landrace) gilts, equally divided into two batches, were used. Gilts were born at the organic farm of the Pig Research Centre of Wageningen Livestock Research, Raalte, The Netherlands. At 9 weeks of age, 48 healthy gilts per batch were transported to the experimental farm ‘Carus’ of Wageningen University, Wageningen, The Netherlands, where they were housed in eight groups of six unrelated pigs in 5.1 m^2^ pens. The floors of the pens were covered with wood shavings (68 l) and straw (around 1.5 kg). Pens were cleaned every day after which fresh straw and wood shavings (together about 500 g) were added. Food (a standard commercial diet for growing pigs) and water were available ad libitum. Lights were on between 7 a.m. and 7 p.m. Pigs could be individually recognized by an ear tag and a number sprayed (Raidex, Kommer Biopharm B.V., Heiloo, The Netherlands) on their backs. The study was approved by the Animal Care and Use Committee of Wageningen University.

### Experimental setup

Pigs were kept in groups of six. Two pigs of each pen, the training pigs, were trained over a period of about 3 weeks to anticipate and experience a positive or negative treatment using a within-subjects design. Thereafter, the training pigs were joined by two non-trained, naive pen mates during anticipation and experience of the treatments to test for emotional contagion. Subsequently, the effect of oxytocin, administered to the non-trained, naive pen mates, on emotional contagion during either a positive situation (half of the pigs) or a negative situation (the other half of the pigs) was studied. Naive pen mates were their own controls for oxytocin versus placebo administration. Finally, two other pigs from each pen, different from the training pigs and naive pigs, were used to test the effect of oxytocin administration per se (referred to as the control pigs). This is presented in Table 1.

**Table 1:** Overview of the experiment split up for the training pigs, naive pigs and control pigs

Week	Test day^a^	Training pigs	Pen mates of training pigs	
			Naive pigs	Control pigs
1	1–7	Training to associate one cue with a positive and another cue with a negative treatment		
2	8–12	Training		
3	15–18	Training		
	19	Training	Habituation to cues and test room	
4	22	Training	Habituation to cues and test room	
	23	Training	Habituation	
	24	Training	Habituation	
	25	Training + habituation for test day 26	Habituation for test day 26	
	26	Test for emotional contagion		
5	29	Training (Bach cue only)	Habituation to cue and test room	
	30	Training		Effect oxytocin on behavior itself
	31	Training		Effect oxytocin on behavior itself
	32	Test for emotional contagion after intranasal oxytocin or placebo administration to the naive pigs		
	33	Test for emotional contagion after intranasal oxytocin or placebo administration to the naive pigs		

^a^Test days 20 and 21 and 27 and 28 were two test-free weekends

Anticipatory behavior in the training pigs was induced using Pavlovian conditioning in which an initially neutral stimulus (conditioned stimulus, CS) was repeatedly followed by a supposedly positive or negative treatment (unconditioned stimulus, US). The supposedly positive treatment consisted of four min access in pairs to a compartment (15.5 m^2^) containing about five kg of straw, 350 l of peat and eight chocolate raisins hidden in the substrate. The supposedly negative treatment consisted of four min social isolation in a much smaller and empty compartment (2.3 m^2^) combined with other negative, unpredictable handlings (see next section). As conditioned stimuli, two auditory cues were used: a repetition of 12 s of piano music from Bach and a repetition of 11 s of a military march (both pieces of music are part of the auditory files of Microsoft PowerPoint 2010). For half of the pens, the piano piece announced the supposedly positive treatment and the military march the supposedly negative treatment. This was the other way around for the other half of the pens. The auditory cue started when both pigs were present in the anticipation compartment with the door closed and ended at the end of the four min treatment period. The cue was played during the treatment as well to increase the likelihood of associating a particular cue with a particular treatment.

**Fig. 1:**
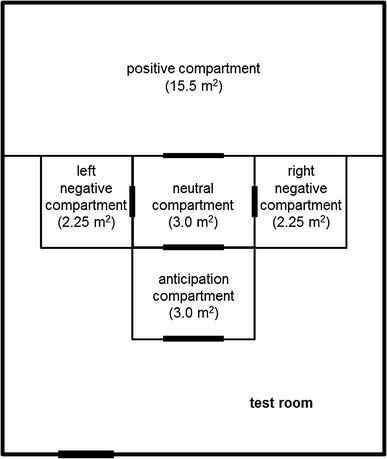
A layout of the test room. The *thickened lines* indicate the position of the doors. The doors are named according to which compartment they gave entrance to. The route from entering the test room to entering the anticipation compartment was separated from the rest of the test room with wooden partitions. The test room was 3.3 m high and the compartment walls and doors were 1.4 m high, except for the neutral compartment door that was 1 m high. Compartments were made of 15-mm-thick chipboard. During the test for emotional contagion, training and naive pigs could therefore hear and smell but not see each other

The experimental setup (Fig. 1) was located in a test room and consisted of five compartments: an anticipation compartment, a positive compartment, two negative compartments and a compartment where the non-trained pen mates stood during the test for emotional contagion (from here on referred to as the neutral compartment). From the anticipation compartment, the training pigs could go to the positive or negative compartments via the neutral compartment after an experimenter had opened the corresponding doors. The positive and negative compartments were not adjacent to the anticipation compartment to prevent the training pigs to see and touch the doors of those compartments during anticipation, because this could have led to differences in ‘door investigatory behaviors’ and ‘head-oriented behaviors’ between positive and negative anticipation (Reimert et al. 2013). However, the aim is to find differences in behaviors indicative of emotional states between positive and negative anticipation, and therefore, the extra, neutral compartment was added between the anticipation and the treatment compartments in this study. Cameras were fixed onto the setup to make video recordings that were analyzed later.

### Training procedure of training pigs

The training procedure lasted about 3 weeks. During these 3 weeks, each pair of training pigs was subjected to two trials per day, one in the morning and one in the afternoon, in one of which they were exposed to the cue followed by the supposedly positive treatment and in the other to the cue followed by the supposedly negative treatment, except for four training days. On these days, the same treatment was given both in the morning and in the afternoon, in a balanced way. We did this, so that the training pigs could not learn which treatment would be given in the afternoon based on the treatment given in the morning and, thus, already start to show anticipatory behavior in the home pen. There were at least 3 h between the two daily trials for each pen. The order in which the training pigs were trained, and the order of positive and negative treatments on a day was randomized for pen and day throughout the entire training period, but in such a way that all pens experienced the positive and negative treatments the same number of times. After each trial, compartments were cleaned (i.e., defecations on the floor of the anticipation and negative compartments were removed after which the floors (and walls) were scrubbed using a cleaning brush, water and cleaning agent and subsequently dried with a towel, and soiled straw and peat were removed from the positive compartment).

During each trial, the two training pigs of each pen were brought into the anticipation compartment after which the cue started. The length of the anticipation period was gradually increased every 2 days from 5 s on the first test day to a maximum of 35 s. With a relatively short anticipation period on the first test day, pigs would likely easily learn the association between the CS and the UCS. The anticipation period was subsequently increased to a time period (i.e., 35 s), we believed sufficient to be able to measure behavioral responses, but not so long to disrupt the association learned. By doing this gradually, the pigs would be habituated to the 35 s step by step. When the cue signaled the supposedly positive treatment, one experimenter entered the anticipation compartment directly after the end of the anticipation period and guided the pair to the positive compartment while another experimenter opened the corresponding doors. The door of the positive compartment was closed as soon as the pair had entered it. After four min in this compartment, the pair was brought back to its home pen. When the cue signaled the supposedly negative treatment, an experimenter entered the anticipation compartment directly after the anticipation period had ended and guided each pig into one of the negative compartments while another experimenter opened the corresponding doors. The door was closed as soon as a pig had entered the negative compartment. After four min in this compartment, the training pigs were brought back to their home pen. In addition to social isolation, other negative handlings were carried out in an unpredictable way during the negative treatment. This was, because pigs usually quickly habituate to stressors and unpredictable negative situations have been shown to be aversive to animals (Weiss 1970; Harding et al. 2004; Koolhaas et al. 2011). On test days 2, 4, 9, 12, 16, 19, 22 and 25, a person (not one of the experimenters) entered one of the negative compartments either directly or 2 min after the start of the treatment and restrained the pig there with a nose sling for 15 s. Thereafter, the same handling was done to the pig in the other negative compartment. On test days 3, 5, 8, 11, 17 and 18, a person also entered one of the negative compartments either directly or one or two min after the start of the treatment but now only threatened to restrain the pig in that compartment by approaching the pig and showing the nose sling to the pig but not restraining the pig. Thereafter, again, the same handling was done to the pig in the other negative compartment. On test days 6 and 10, air from a noisy vacuum cleaner was blown for 15 s into both negative compartments at floor level at one min after the start of the treatment, and on test days 7 and 15, two balloons, one at the level of each compartment, were simultaneously punctured with a needle at one min after the start of the treatment. On test days 1, 23, 24 and 26, no additional handlings were carried out. Assignment of compartments (left or right) and (start of the) negative handlings were all balanced for the different test days and over the total training period. Days 13, 14, 20 and 21 were 2 weekends during which pigs were not trained.

The behaviors of the training pigs during anticipation and during the experience of positive and negative treatments on test days 23 and 24 were considered as ‘normal’ behaviors expressed during a particular training trial and were used to compare with their behavior on test day 26, when two non-trained pen mates were also present during anticipation and the treatments which allowed testing for emotional contagion (see “Test for emotional contagion” section).

### Habituation procedure of the pen mates

Before testing for emotional contagion on test day 26, the four pen mates of each pair of training pigs were habituated to the test room and to the piano music and military march (i.e., the CSs), but were not given access to the treatments. Two of these pigs will later on join the training pigs in the test room to test for emotional contagion (see “Test for emotional contagion” section). These two are from here on referred to as naive pigs or naive pen mates as they are, with regard to the training pigs, naive to the treatments. The other two will later on be used to test whether oxytocin has an effect on behavior in itself, irrespective of the treatment of the training pigs (see “Test for the effect of oxytocin on emotional contagion” section). These two are from here on referred to as control pigs.

Habituation started on test day 19 by bringing these four pigs to the anticipation compartment, and after the door was closed, one of the cues started. After 35 s, pigs were guided by an experimenter to the neutral compartment, while another experimenter opened the door for them. Subsequently, the four pigs spent two min in the neutral compartment after which the cue ended and they were brought back to their home pen. Thereafter, it was the turn of the next four pigs. Similar to the training pigs, these four pigs also had a morning and afternoon trial, one with the Bach cue and one with the military march cue, matching the positive and negative cue of their trained pen mates. The four pigs were habituated in this way for two test days. In the following two test days, a similar procedure was carried out, but now with only two of the four pigs, i.e., the naive pigs, and the duration in the neutral compartment was set to four min. The order in which these pigs were habituated and which cue was given in the morning and which in the afternoon trial were randomized, but balanced for pen and day on these four test days. There were at least 3 h between the two daily trials for each pen.

The behaviors of the two naive pigs in the anticipation and neutral compartment on the last two habituation days, test days 23 and 24, were used to compare with their behavior in the same situation on test day 26, i.e., the emotional contagion test day.

On test day 25, the two training and two naive pigs of each pen were brought once to the anticipation and neutral compartment to habituate them to the presence of the other two pigs and to being split up. After 35 s in the anticipation compartment, the door to the neutral compartment was opened and an experimenter came in to separate the training from the naive pigs without actually putting the training pigs in either the positive or negative compartment. Thereafter, the four pigs were brought back to their home pen. No cue was given during the 35 s in the anticipation compartment. In this way, any disturbance of the company of these two naive pen mates in the training pigs and vice versa on test day 26 may be reduced.

### Test for emotional contagion

In the morning and afternoon of test day 26, the training pigs and their two naive pen mates were brought to the anticipation compartment where a cue was given for 35 s, after which the training pigs were exposed to the corresponding positive or negative treatment for four min. The naive pen mates stayed in the neutral compartment during these four min. After the four min, all four pigs were brought back to their home pen. Half of the pens were exposed to the supposedly positive treatment in the morning and to the supposedly negative treatment in the afternoon, and for the other pens, this was the other way around.

### Test for the effect of oxytocin on emotional contagion

In the week after test day 26 (a Friday), the test for emotional contagion was repeated, but this time the naive pen mates received a dose of oxytocin 30 min before they were brought to the test room together with the training pigs. To avoid that the naive pen mates started to anticipate themselves, we kept the number of test trials to a minimum by continuing with only one cue (i.e., the Bach cue) for all pens. So from here on, half of the training pigs only experienced the positive treatment and the other half only the negative treatment.

In the morning of the first 3 days of this week, the training pigs of each pen went through a regular training trial to keep the association between the Bach cue and the subsequent treatment. After the training trials on the first day of this week, the four pen mates of the training pigs were reminded of the test room in the same way as described above, but also with just the Bach cue. After the training trials on the second and third day (i.e., test days 30 and 31) of this week, the effect of oxytocin on behavior in itself was studied with the control pigs. On the first of these 2 days, therefore, half of the pairs received a single dose of 24 IU of oxytocin (VWR International B.V., Amsterdam, The Netherlands) 30 min before they were brought to the test room and the other half received a placebo. For the oxytocin, this was done by diluting 50 μg of oxytocin in 0.5 ml of 0.9 % saline and administering 0.25 ml in each nostril of each pig using a Mucosal Atomizer Device (MAD 300, Vandeputte Medical Nederland B.V., Nieuwegein, The Netherlands) connected to a 1-ml syringe (Rault et al. 2013). The placebo consisted of 0.5 ml of 0.9 % saline which was administered in the same way as the oxytocin solution. Subsequently, the pair of control pigs was brought to the test room 30 min later, and the same procedure as described above was carried out, meaning that control pigs were brought to the test room without training pigs. On the second day, the pairs that received oxytocin the day before were now given the placebo and vice versa.

On the last two test days of this week (test days 32 and 33), the effect of oxytocin on emotional contagion was tested. Hereto, the same procedure for the oxytocin and placebo administration was used as described above, except that here the pen mates, i.e., the naive pigs, were used that were also used on test day 26. Further, the same procedure as described for test day 26 was followed, meaning that both naive and training pigs were brought to the test room.

### Behavioral analyses

The ethogram in Table 2 was used for scoring behaviors displayed in the anticipation compartment on test days 9, 12, 17, 18, 23, 24, 26 and 30–33 by all pigs and during the positive and negative treatments by the training pigs and in the neutral compartment by the naive pen mates on test days 23, 24, 26 and 30–33 (see Online Resource 1 for more information). The vocalizations in Table 2 were scored as events during the actual trials (i.e., live) and were scored as a total of two pigs on test days 9–24, 30 and 31 and as a total of four pigs on test days 26, 32 and 33, because it was not possible to identify them per individual pig. Defecating on these days was scored by counting the number of fecal droppings, and urinating was scored as being present or absent in each compartment after every trial. The other behaviors in Table 2 were scored as states from the video recordings using focal sampling and continuous recording with the Observer XT 10 software of Noldus Information Technology B.V., Wageningen, The Netherlands. Not all these behaviors were scored for each pig in each situation, either because that was not possible (e.g., exploring anticipation door for the training pigs during the treatment) or because behaviors were regarded to be relevant for one situation only [e.g., head postures were only scored in the anticipation compartment, because the number of transitions between both head postures could be indicative for hyperactive behavior which has been associated with anticipation of positive stimuli (Moe et al. 2011; Spruijt et al. 2001)].

**Table 2:** Ethogram used to score the behaviors of both the training pigs and naive pen mates in the anticipation compartment on test days 9, 12, 17, 18, 23, 24, 26 and 30–33 and during the treatments (in the positive and negative compartment for the training pigs and in the neutral compartment for the naive pen mates) on test days 23, 24, 26 and 30–33

	Description
Behavior	
Standing alert	Standing motionless with whole body and head fixed
Escape attempts	Jumping in air or against the wall or door of a compartment
Play	Running, gamboling, pivoting or playing with straw by shaking head
Urinating (event)	Urinating
Defecating (event)	Defecating
Exploring anticipation door^a^	Sniffing, nosing or rooting the door of the anticipation compartment
Exploring neutral door^b^	Sniffing, nosing or rooting the door of the neutral compartment
Exploring positive door^c^	Sniffing, nosing or rooting the door of the positive treatment compartment
Exploring negative doors^c^	Sniffing, nosing or rooting the door of one of the negative treatment compartments
Ears postures	
Ears front	Both ears directed to the front
Ears back	One or both ears directed backwards
Tail postures	
Tail in curl	Tail coiled up in a curl on top of the body
Tail wagging	Tail swinging in any direction, but mostly from side to side
Tail low	Tail hanging down against the body
Head postures^a^	
Head up	Head directed forward or actively up
Head down	Head directed downwards or to the floor of the compartment
Head orientation^a^	
Head to anticipation door	Head oriented to the door of the anticipation compartment
Head to neutral door	Head oriented to the door of the neutral compartment
Vocalizations (events)	
Low-pitched vocalizations	Short or long grunts
High-pitched vocalizations	Screams, squeals or grunt squeals
Barks	A low tone that sounds like “woof”

Behaviors were scored as states unless indicated otherwise

^a^These behaviors were only scored when the pigs were in the anticipation compartment

^b^This behavior was not scored for the training pigs during the treatments

^c^These behaviors were not scored when the pigs were in the anticipation compartment and exploring the negative doors and exploring the positive door were not scored for the training pigs when they were in the positive and negative treatments, respectively

### Statistical analyses

SAS (SAS 9.2, SAS Institute Inc., Cary, NC, USA) was used for all statistical analyses. Preliminary analyses showed that the behavior of the training pigs on test days 23, 24 and 26 differed substantially between the positive and negative treatments. However, their behavior on these and earlier test days in the anticipation compartment showed only subtle differences between anticipation of positive and negative stimuli (data not shown), and the type of behavioral differences found between anticipation of negative versus positive stimuli seemed specific for the individual training pigs involved, i.e., there was no general, clear pattern of behaviors differing between positive and negative anticipation in all training pigs. In addition, the behavior of the naive pen mates on test day 26 seemed to indicate that they were (emotionally) affected by the training pigs during the two treatments, but not during anticipation. Therefore, we decided to omit the results of the training and naive pigs in the anticipation compartment and thus only present the results of the training and naive pigs during the positive and negative treatments.

#### Emotional contagion without a possible effect of intranasal oxytocin

Before analyses, the behaviors of the training pigs in the different treatments (i.e., positive or negative) were averaged per pen. Subsequently, the behaviors of the pairs during the treatments were also averaged over test days 23 and 24 to have one representative value of the behaviors of a pair of pigs during the positive and negative treatments. Preliminary analyses showed no effect of cue (i.e., Bach or military march) on the behaviors of the training pigs during the treatments. This factor was, therefore, not included in the final models. Behaviors were analyzed with three separate analyses: (1) differences between the treatments were investigated in the situation without naive pen mates present (i.e., using the pen averages of the behaviors expressed in the treatments over test days 23 and 24), (2) differences between treatments were investigated in the situation with naive pen mates present (i.e., using the pen averages of the behaviors expressed in the treatments on test day 26) and (3) differences between treatments were investigated by using a model that included both situations. For the first and second analyses, a general linear model (GLM) was used with treatment (i.e., positive or negative) and batch (i.e., batch 1 and 2) as fixed effects, and for the third analysis, a GLM was used with treatment, situation (without or with naive pen mates present), their interaction and batch as fixed effects. The behaviors that were far from normally distributed (e.g., pigs generally urinated once or not at all) were transformed into a 0–1 variable on pen level and were analyzed with a generalized linear model with a logit link and binary distribution and with the same fixed effects as used in the GLM. When those behaviors also did not occur during either the negative or the positive treatment (e.g., no play behavior was observed in the negative treatment), they were analyzed with a Fisher’s exact test for treatment for the first and second situations separately and over both situations and with a Fisher’s exact test for situation within each treatment and over the two treatments.

For the behaviors of the naive pen mates, similar (statistical) procedures were followed. For the first analysis (i.e., the situation without training pigs present in the test room), the fixed effect treatment was, however, changed into cue (i.e., Bach or military march) as naive pigs were exposed to two different cues, but not to the actual treatments. Cue did not affect any of the behaviors (see Table 3). Therefore, differences in behavior in the situation with training pigs present in the positive or negative treatment were analyzed using a GLM with treatment (i.e., average of both cues, positive or negative) and batch as fixed effects.

**Table 3:** Behavior of the naive pen mates in the neutral compartment of the test room without training pigs present but with two different cues

	Without training pigs present		C^1^
	Bach	Military march	
Behavior			
Standing alert (% of time)	5.6 ± 1.4	3.6 ± 1.0	NS
Escape attempts (freq.)	0.9 ± 0.3	0.5 ± 0.2	NS
Urinating (% of pens)	75.0	68.8	NS
Defecating (freq.)	4.2 ± 0.3	3.8 ± 0.3	NS
Exploring neutral door (% of time)	7.0 ± 1.0	6.0 ± 1.2	NS
Exploring positive door (% of time)	2.6 ± 0.9	1.3 ± 0.3	NS
Exploring negative door (% of time)	2.2 ± 0.3	2.3 ± 0.3	NS
Ear posture			
Ears back (% of time)	3.8 ± 0.8	3.7 ± 1.0	NS
Vocalizations (voc.)			
Low-pitched voc. (freq.)	10.2 ± 2.5	11.0 ± 2.3	NS
High-pitched voc. (freq.)	6.2 ± 3.2	5.9 ± 2.4	NS
Barks (% of pens)	0	6.3	NS

^1^Significance of effect of cue (C) is indicated: NS *P* ≥ 0.10

#### Emotional contagion with a possible effect of intranasal oxytocin

Preliminary analyses showed no effect of oxytocin on the behavior of the control pigs in the test room on test days 30 and 31 (see Table 4). Moreover, order (i.e., receiving oxytocin first and then the placebo or vice versa) also did not affect the behavior of pigs on these test days and test days 32 and 33. Order was, therefore, not included in the final models.

**Table 4:** Behavior of the control pigs in the neutral compartment of the test room 30 min after receiving an intranasal administration of oxytocin or a placebo

	Oxytocin	Placebo	A^1^
Behavior			
Standing alert (% of time)	13.6 ± 2.0	12.8 ± 1.5	NS
Escape attempts (% of pens)	6.3	6.3	NS
Urinating (% of pens)	50.0	68.8	NS
Defecating (freq.)	4.3 ± 0.3	3.9 ± 0.4	NS
Exploring neutral door (% of time)	7.8 ± 2.0	8.0 ± 1.2	NS
Exploring positive door (% of time)	4.2 ± 0.9	5.5 ± 1.3	NS
Exploring negative door (% of time)	1.8 ± 0.4	1.5 ± 0.3	NS
Ear posture			
Ears back (% of time)	4.8 ± 1.3	4.5 ± 1.3	NS
Tail postures			
Tail in curl (% of time)	98.5 ± 1.0	97.9 ± 1.2	NS
Tail wagging (% of time)	0.6 ± 0.5	1.1 ± 0.9	NS
Tail low (% of time)	0.9 ± 0.8	1.0 ± 0.6	NS
Vocalizations (voc.)			
Low-pitched voc. (freq.)	4.5 ± 1.3	2.5 ± 1.2	NS
High-pitched voc. (freq.)	0.2 ± 0.1	0.3 ± 0.2	NS
Barks (% of pens)	0	0	NS

^1^Significance of effect of intranasal administration (A) is indicated: NS *P* ≥ 0.10

The behaviors of the training and naive pigs on test days 32 and 33 were analyzed on pen level with a mixed linear model with treatment (i.e., positive or negative), intranasal administration (i.e., oxytocin or a placebo), their interaction and batch as fixed effects and pen nested within treatment and batch as a random effect. Similar to before, behaviors that were far from normally distributed were transformed into a 0–1 variable on pen level and analyzed with a generalized linear model with a logit link and binary distribution and with the same fixed effects as used in the mixed linear model or with a Fisher’s exact test for treatment, intranasal administration and treatment within each administration when the behavior also did not occur during either the negative or the positive treatment.

As the two ear postures are complementary to each other, only the percentage of time ears back are presented as this posture has been associated with a negative emotional state (Reimert et al. 2013). For the naive pen mates, play behavior and tail postures were not statistically analyzed, because play did not occur and the tail was almost 100 % of the time in a curl (“tail in curl”: 99.7 ± 0.1 % of time; “tail wagging”: 0.1 ± 0.1 % of time; “tail low”: 0.1 ± 0.1 % of time). The behaviors analyzed with the GLM or mixed linear model were expressed as percentage of time or as absolute frequencies and the behaviors analyzed with the Fisher’s exact test or generalized linear model as percentage of pens that showed this behavior. For the GLM and mixed models, skewed residuals were normalized if needed using arcsine square root and square root transformations for proportions and frequencies, respectively, and significant interactions were further explored with post hoc pairwise comparisons using the differences of the least square means.

## Results

### Behavior of training and naive pigs without intranasal oxytocin administration

#### Training pigs

In the situation without the presence of the naive pen mates, treatment affected all behaviors of the trainings pigs except the tail posture “tail low” which did not differ between the positive and negative treatments (Table 5). Play behavior occurred and barks were heard during the positive treatment only. In addition, training pigs wagged their tail far more during the positive treatment than during the negative treatment. Escape attempts occurred and high-pitched vocalizations were heard during the negative treatment only. Also, training pigs showed more standing alert behavior, were more likely to urinate and defecate, showed more exploring of the compartment door, had their ears more in a backwards posture and their tail more in a curl posture and produced more low-pitched vocalizations during the negative treatment than during the positive treatment. In the situation with two naive pen mates present in the neutral compartment, treatment affected the behaviors of the trainings pigs similar to the situation where training pigs were tested alone (Table 5).

**Table 5:** Behavior of the training pigs during positive and negative treatments in two situations: without the presence of two naive pen mates and in the presence of two naive pen mates in the test room

	Without naive pigs present			With naive pigs present		Effects^1^		
	Positive	Negative	T^2^	Positive	Negative	T^2^	S	TS
Behavior								
Standing alert (% of time)	0.3 ± 0.1	32.9 ± 3.1	***	2.8 ± 1.2^a^	49.0 ± 4.6^c^	***	**	*
Escape attempts (% of pens)^3^	0	62.5	***	0	31.3	*	NS	–
Play (% of pens)^3^	100	0	***	93.8	0	***	NS	–
Urinating (% of pens)^3^	6.3	93.8^g^	***	0	62.5^h^	***	NS	–
Defecating (freq.)	0.7 ± 0.2	4.7 ± 0.5	***	0.7 ± 0.3	4.5 ± 0.4	***	NS	NS
Exploring treatment door (% of time)	0.5 ± 0.1	3.4 ± 0.7	***	0.4 ± 0.2	2.3 ± 0.6	***	^+^	NS
Ear posture								
Ears back (% of time)	1.9 ± 0.7	17.3 ± 4.7	***	1.3 ± 0.5^a^	7.3 ± 2.0^c^	**	*	^+^
Tail postures								
Tail in curl (% of time)	87.3 ± 3.5	99.8 ± 0.2	***	93.1 ± 2.4^c^	99.2 ± 0.7^b^	**	NS	^+^
Tail wagging (% of time)	12.3 ± 3.4	0.1 ± 0.0	***	6.7 ± 2.3^c^	0.2 ± 0.1^b^	***	^+^	^+^
Tail low (% of time)	0.4 ± 0.2	0.1 ± 0.1	NS	0.3 ± 0.2	0.6 ± 0.5	NS	NS	NS
Vocalizations (voc.)								
Low-pitched voc. (freq.)	0.2 ± 0.2	24.8 ± 2.9	***					
High-pitched voc. (% of pens)	0	50.0	**					
Barks (% of pens)	87.5	0	***					

Means with different superscript letters differ significantly (a/b/c: *P* < 0.05; g/h: *P* < 0.1)

^1^Significance of effects of treatment (T), situation (S) and their interaction (TS) is indicated: *** *P* < 0.001; ** *P* < 0.01; * *P* < 0.05; ^+^ *P* < 0.10; NS *P* ≥ 0.10; – no statistical analysis performed

^2^These treatment effects belong to the first and second situations, respectively. Treatment effects over both situations were equal to the situation without naive pigs present

^3^The effect of situation within treatment was significant for urinating within the negative treatment, but not within the positive treatment nor for escape attempts and play

When comparing both situations, training pigs generally tended to explore the door of the treatment compartment less when their naive pen mates were present in the neutral compartment than without their presence (Table 5). In addition, a significant interaction effect was found for standing alert and a tendency for an interaction effect for ears back and the tail postures “tail in curl” and “tail wagging” (Table 5). Post hoc pairwise comparisons revealed that training pigs spent more time standing alert but had their ears less backwards and tended to urinate less during the negative treatment with two naive pen mates present than during the same treatment without their presence (Table 5). During the positive treatment, training pigs had their tails in a curl more frequently and wagged their tails less when their two naive pen mates were present than during the same treatment without their presence (Table 5). Training pigs also tried to escape less from the negative treatment compartment when their naive pen mates were present in the neutral compartment than without their presence, but that was not significant (Table 5).

#### Naive pen mates

The behavior of the naive pen mates in the neutral compartment of the test room was not affected by hearing Bach music or a military march (Table 3). There were differences, however, between the situation where naive pigs were tested alone versus the situation with training pigs present in either the positive or the negative treatment compartment for standing alert, exploring of the compartment doors and ears back (Table 6). Post hoc analysis showed that the naive pen mates spent more time standing alert when the training pigs were in the negative treatment than when the training pigs were in the positive treatment or in the situation without training pigs present in the test room (Table 6). Furthermore, naive pen mates spent more time exploring the door of the compartment that held the training pigs during both treatments, but they spent more time exploring the door of the neutral compartment in the situation without the training pigs present compared to the situation with training pigs present in the positive treatment. In addition, naive pigs tended to spend more time exploring the door of the neutral compartment in the situation without the training pigs present compared to the situation with training pigs present in the negative treatment (Table 6). Moreover, naive pen mates tended to have their ears more backward when the training pigs were in the negative treatment than when the training pigs were in the positive treatment and they had their ears more backward when the training pigs were in the negative treatment than in the situation without training pigs present in the test room (Table 6).

**Table 6:** Behavior of the naive pen mates in the neutral compartment of the test room in three situations: without training pigs present and with training pigs present in the positive or negative treatment compartments

	Without training pigs present	With training pigs present		T^1^
		Positive	Negative	
Behavior				
Standing alert (% of time)	4.6 ± 1.2^a^	3.8 ± 0.8^a^	10.7 ± 1.6^b^	***
Escape attempts (freq.)	0.7 ± 0.3	0.4 ± 0.2	0.4 ± 0.2	NS
Urinating (% of pens)	87.5	62.5	43.8	NS
Defecating (freq.)	4.0 ± 0.3	3.8 ± 0.3	3.9 ± 0.3	NS
Exploring neutral door (% of time)	6.5 ± 1.0^ay^	3.7 ± 0.7^bz^	4.3 ± 0.8^z^	^+^
Exploring positive door (% of time)	2.0 ± 0.5^a^	6.4 ± 1.9^b^	2.6 ± 0.7^a^	*
Exploring negative door (% of time)	2.2 ± 0.2^a^	2.1 ± 0.4^a^	3.6 ± 0.6^b^	*
Ear posture				
Ears back (% of time)	3.8 ± 0.9^ay^	4.5 ± 1.0^y^	7.2 ± 1.4^bz^	^+^

Means with different superscript letters differ significantly (a/b: *P* < 0.05; y/z: *P* < 0.10)

^1^Significance of effect of treatment (T) is indicated: *** *P* < 0.001; * *P* < 0.05; ^+^ *P* < 0.10; NS *P* ≥ 0.10

#### Vocalizations in the situation with four pigs in the test room

Vocalizations were not compared between both situations, because vocalizations were scored as a total of two pigs in one and as a total of four pigs in the other situation. In the situation with the training pigs in one of the treatment compartments and their naive pen mates in the neutral compartment, more low- and high-pitched vocalizations were recorded with the training pigs in the negative treatment compartment than in the positive treatment compartment (low-pitched vocalizations: 33.6 ± 3.6 vs. 7.9 ± 2.5, *P* < 0.001 and high-pitched vocalizations: 10.7 ± 3.5 vs. 2.9 ± 1.4, *P* < 0.001). In contrast, more barks were heard with the training pigs in the positive treatment compartment than in the negative treatment compartment (56.3 vs. 6.3 % of pens, *P* < 0.01).

### Behavior of training and naive pigs with intranasal oxytocin administration

#### Training pigs

Irrespective of whether their naive pen mates received an intranasal administration of oxytocin or a placebo, training pigs still only played during the positive treatment and wagged their tails more during this treatment than during the negative treatment (Table 7). During the negative treatment, training pigs still spent more time standing alert, were more likely to urinate and had their tails more in a curl than during the positive treatment. No main effect of treatment was found for escape attempts, exploring of the treatment door, and ears backward (Table 7).

**Table 7:** Behavior of the training pigs during positive and negative treatments in the presence of their naive pen mates who received an administration of oxytocin or a placebo 30 min before they went to the test room with the training pigs

	Positive treatment		Negative treatment		Effects^1^		
	Oxytocin	Placebo	Oxytocin	Placebo	T	A	TA
Behavior							
Standing alert (% of time)	3.5 ± 2.1	4.3 ± 3.0	33.9 ± 3.2	34.3 ± 4.0	***	NS	NS
Escape attempts (% of pens)^2^	0	12.5	0	0	NS	NS	–
Play (% of pens)^2^	100^g^	100^g^	0^h^	0^h^	***	NS	–
Urinating (% of pens)^2^	0^g^	0^g^	75.0^h^	87.5^h^	***	NS	–
Defecating (freq.)	0.3 ± 0.2^a^	0.3 ± 0.1^a^	4.1 ± 0.4^b^	5.1 ± 0.3^c^	***	*	*
Exploring treatment door (% of time)	3.2 ± 1.2^a^	1.1 ± 0.6^b^	2.3 ± 0.5^ab^	3.2 ± 0.4^b^	NS	NS	*
Ear posture							
Ears back (% of time)	10.3 ± 4.0	13.5 ± 2.7	6.1 ± 1.6	6.8 ± 1.3	NS	NS	NS
Tail postures							
Tail in curl (% of time)	85.0 ± 5.9	82.0 ± 7.0	99.9 ± 0.1	100 ± 0.0	**	NS	NS
Tail wagging (% of time)	11.6 ± 4.8	9.2 ± 3.9	0	0.0 ± 0.0	**	NS	NS
Tail low (% of time)	3.4 ± 2.2^a^	8.8 ± 4.2^b^	0.1 ± 0.1^a^	0^a^	^+^	^+^	*

Means with different superscript letters differ significantly (a/b/c: *P* < 0.05; g/h: *P* < 0.01)

^1^Significance of effects of treatment (T), intranasal administration (A) and their interaction (TA) is indicated: *** *P* < 0.001; ** *P* < 0.01; * *P* < 0.05; ^+^ *P* < 0.10; NS *P* ≥ 0.10; – no statistical analysis performed

^2^The effect of treatment within the oxytocin or placebo administration was not significant for escape attempts, but was significant for play (*P* < 0.001) and for urinating (*P* < 0.01)

Defecating was and “tail low” tended to be affected by treatment, administration and their interaction (Table 7). Post hoc analysis showed that training pigs were less likely to defecate during the positive treatment than during the negative treatment, but were also less likely to defecate during the negative treatment when their naive pen mates had received oxytocin compared to a placebo (Table 7). Furthermore, the tail of the training pigs was most frequently “low” during the positive treatment and with a placebo given to their naive pen mates compared to the other situations (Table 7). Exploring the treatment door was also affected by the interaction between treatment and administration. Post hoc analysis showed that training pigs spent less time exploring the door of the treatment compartment during the positive treatment when their naive pen mates had received a placebo than during the positive treatment when their naive pen mates had received oxytocin. In addition, they tended to spend less time exploring the door of the treatment compartment during the positive treatment when their naive pen mates had received a placebo than during the negative treatment when their naive pen mates had received a placebo (Table 7).

#### Naive pen mates

Irrespective of whether the naive pen mates received oxytocin or a placebo, they spent more time standing alert when the training pigs were in the negative treatment than when the training pigs were in the positive treatment (Table 8). In addition, naive pen mates also tended to have their ears more backward when the training pigs were in the negative treatment than when the training pigs were in the positive treatment (Table 8). On the other hand, naive pen mates spent more time exploring the door of the positive treatment compartment when the training pigs were in the positive treatment than when training pigs were in the negative treatment (Table 8). A significant interaction effect between treatment and administration was found for exploring the neutral door, which was during the negative treatment performed more by the placebo-treated pigs and during the positive treatment more by the oxytocin-treated pigs, although post hoc analysis revealed no differences between the treatment groups (Table 8).

**Table 8:** Behavior of the naive pen mates in the neutral compartment of the test room 30 min after receiving an intranasal administration of oxytocin or a placebo and during a positive or negative treatment experienced by the training pigs

	Positive treatment		Negative treatment		Effects^1^		
	Oxytocin	Placebo	Oxytocin	Placebo	T	A	TA
Behavior							
Standing alert (% of time)	5.5 ± 2.1	9.0 ± 2.6	13.8 ± 2.0	14.4 ± 2.8	*	NS	NS
Escape attempts (% of pens)^2^	0	0	25.0	0	NS	NS	–
Urinating (% of pens)	37.5	75.0	62.5	75.0	NS	NS	NS
Defecating (freq.)	3.0 ± 0.5	2.9 ± 0.6	3.9 ± 0.6	3.6 ± 0.4	NS	NS	NS
Exploring neutral door (% of time)	6.1 ± 3.0	3.2 ± 1.4	5.1 ± 1.2	7.2 ± 2.2	NS	NS	*
Exploring positive door (% of time)	35.0 ± 10.4	32.3 ± 11.1	5.4 ± 1.4	4.2 ± 1.4	*	NS	NS
Exploring negative door (% of time)	0.8 ± 0.4	0.9 ± 0.2	1.0 ± 0.3	1.2 ± 0.3	NS	NS	NS
Ear posture							
Ears back (% of time)	5.8 ± 1.6	6.1 ± 4.2	10.0 ± 1.9	9.9 ± 1.7	^+^	NS	NS

^1^Significance of effects of treatment (T), intranasal administration (A) and their interaction (TA) is indicated is indicated: * *P* < 0.05; ^+^ *P* < 0.10; NS *P* ≥ 0.10; – no statistical analysis performed

^2^The effect of treatment within the oxytocin or placebo administration was also not significant

#### Vocalizations

The four pigs together produced more low-pitched vocalizations and tended to produce more high-pitched vocalizations during the negative treatment than during the positive treatment of the training pigs (low-pitched vocalizations: 25.5 ± 4.1 vs. 3.6 ± 1.2, *P* < 0.01 and high-pitched vocalizations: 11.8 ± 4.8 vs. 0.2 ± 0.1, *P* < 0.10). In contrast, barks were only heard during the positive treatment of the training pigs (62.5 vs. 0 % of pens, *P* < 0.001). Moreover, more low-pitched vocalizations were produced when the naive pen mates were given oxytocin than a placebo (16.4 ± 4.5 vs. 12.7 ± 3.7, *P* < 0.05). No other significant (interaction) effects were found.

## Discussion

The aim of this study was to test whether emotional contagion occurs in pigs during positive and negative treatments and whether oxytocin augments emotional contagion. The results of this study indeed suggest, although subtle, that pigs can be affected by the emotional state of their pen mates. Furthermore, no effect of oxytocin was found on the behavior of the treated naive pigs, but surprisingly the training pigs did behave differently in the treatments when their naive pen mates were given oxytocin or a placebo.

### Emotional contagion without a possible effect of intranasal oxytocin

During the treatments, training pigs showed many behavioral differences. The design of the positive and negative treatments was (partly) based on other studies (see Reimert et al. 2013), and the behaviors displayed in both treatments indeed showed that the positive treatment elicited a positive emotional state [e.g., play behavior (Boissy et al. 2007; Held and Špinka 2011) and barks (Chan and Newberry 2011; Newberry et al. 1988)] and the negative treatment a negative emotional state [e.g., escape attempts and defecations (Mendl and Paul 2004) and high-pitched vocalizations (Manteuffel et al. 2007)] in the training pigs (see also Reimert et al. 2013 for a more comprehensive explanation). This was true not only for the situation without, but also for the situation with two of their pen mates present in the neutral compartment of the test room. However, training pigs stood alert more, but had their ears back less frequently, tended to urinate less and seemed to try to escape the compartment less during the negative treatment with two of their naive pen mates present than without their presence. These differences suggest that the training pigs were overall less negatively affected by the negative treatment when their pen mates were present in the neutral compartment, which could indicate that the training pigs took the presence of their pen mates as social support (Reimert et al. 2013, 2014). On the other hand, training pigs wagged their tails less during the positive treatment with two of their naive pen mates present than without their presence (see Reimert et al. 2013, for a discussion on the tail posture “tail in curl”). The presence of their pen mates might have made the training pigs more vigilant during the positive treatment (percentage of time standing alert was also increased in this situation, although not significantly so) which resulted therefore in less “tail wagging.” The experiment was set up for naive pen mates to become affected by the emotional state of the training pigs, but training pigs may just as well respond to their naive pen mates. Whether it was an actual emotional state of the pen mates or just their presence that caused these changes in the behavior of the training pigs cannot, however, be elucidated from these results.

The naive pen mates of the training pigs did not behave differently when hearing either Bach or a military march which indicates that these cues in themselves did not have an effect on the behavior of the naive pen mates. In addition, it also indicates that any differences seen in their behavior in the situation when training pigs were present in either the positive or negative treatment compartment are likely due to the (emotional state of the) training pigs. During both treatments, naive pigs spent more time exploring the door of the compartment that held the training pigs. This probably indicated that the naive pen mates realized other pigs were present behind the door and wanted to investigate that, but these behaviors do not necessarily indicate that the naive pigs were emotionally affected by the training pigs. Investigation of a door behind which a negative situation takes place may seem odd, but this also occurred in our previous study (Reimert et al. 2013) and may have been a way of the naive pigs to respond to a threatening or dangerous stimulus (Paul et al. 2005) or may have been a form of vigilance behavior (Welp et al. 2004). The naive pen mates also spent more time standing alert and tended to have their ears more backwards during the negative treatment of the training pigs than during the positive treatment of the training pigs or without training pigs present in the test room. As standing alert behavior and ears back have been associated with a negative emotional state (Boissy 1995; Boissy et al. 2011; Paul et al. 2005; Tate et al. 2006), the naive pen mates were thus, just as the training pigs, likely in a negative emotional state during the negative treatment of the training pigs and that suggests that emotional contagion had occurred in this negative situation. Signals by which emotional contagion could have occurred during this situation could have been auditory or olfactory (Amory and Pearce 2000; Vieuille-Thomas and Signoret 1992), but not visual as training and naive pigs could not see each other during the treatments. Caution is warranted, however, because the other behaviors expressed by the naive pen mates do not indicate that emotional contagion had occurred and something other than the (emotional state of) the training pigs could also have caused the differences in standing alert behavior and ears backward (Edgar et al. 2012a). For instance, the naive pigs could have responded with these behaviors to the high-pitched vocalizations produced by the training pigs during the negative treatment, because these vocalizations represented loud noises which made them vigilant and not because they represented a negative emotional state. This seems, however, not likely, because the naive pigs were not unfamiliar with these vocalizations (i.e., high-pitched vocalizations are also occasionally produced in the home pen) and they responded not in this way to the barks, which are also loud noises, during the positive treatment.

In our previous study, the naive pen mates played during the positive treatment of their trained pen mates, but not during the negative treatment experienced by the training pigs. That the naive pigs did not play in the neutral compartment in the present study could have been due to their somewhat negative emotional state during testing. Their frequencies of urinating and defecating, for instance, are in all three situations comparable to the frequencies of the training pigs during the negative treatment, and these behaviors have been associated with a negative emotional state (Mendl et al. 1997; Mendl and Paul 2004). The naive pigs already displayed this negative emotional state on the first day of habituation. Thus, they evaluated the test room as unpleasant already on the first day and persisted in that evaluation until the end of the experiment. In our previous study, naive pigs were also habituated to the test room, but only for 20 s (i.e., the length of the anticipation period) at a time which was perhaps too short to evaluate the test room as negative or positive for that matter. It is not clear, however, why the naive pigs of the present study experienced the first and subsequent habituation trials as negative. Nevertheless, the results of this study, although subtle, do provide evidence for emotional contagion in pigs.

### Oxytocin and emotional contagion

Similar to the situation without intranasal oxytocin administration, the naive pen mates also spent more time standing alert and tended to have their ears backwards more frequently during the negative treatment of the training pigs than during the positive treatment of the training pigs. As the behavior of the training pigs indicates that they were still in a negative emotional state during the negative treatment, these results suggest that emotional contagion had occurred. No effect of oxytocin was found on the behavior of the control pigs, suggesting that any effect of oxytocin on the behavior of the naive pigs is likely due to the (emotional state of) the training pigs. However, oxytocin did not seem to have an effect on the behaviors of the naive pigs and subsequently also not on their emotional state. This was not as expected. The dose used and the time period between administration and testing were chosen, because this dose and time period have been used frequently by other studies, including a pig study, where clear effects of oxytocin on (emotional) behavior were found (Churchland and Winkielman 2012; MacDonald and MacDonald 2010; Rault et al. 2013; Zink and Meyer-Lindenberg 2012). It may be that the dose and/or time period chosen was not appropriate for the present study which subsequently led to these negative results. At present, however, we cannot explain these results.

Training pigs were now only subjected to either the positive or the negative treatment, but their behavior remained fairly consistent with before. Training pigs still only played during the positive treatment and wagged their tails more during the positive treatment than during the negative treatment, whereas training pigs spent more time standing alert and were more likely to urinate and defecate during the negative treatment than during the positive treatment. These differences indicate that training pigs continued to value the positive treatment as positive and the negative treatment as negative. Surprisingly, effects of oxytocin given to the naive pigs were found on the behavior of the training pigs which had not received oxytocin or a placebo themselves. Trainings pigs were namely less likely to defecate during the negative treatment when their naive pen mates had received oxytocin than during the same treatment when their naive pen mates had received a placebo. Moreover, training pigs explored the door of the treatment compartment more during the positive treatment when their naive pen mates had received oxytocin than during the same treatment when their naive pen mates had received a placebo. However, training pigs also explored the door more during the negative treatment when their naive pen mates had received a placebo. Moreover, training pigs had their tails less frequently in the “low” posture during the positive treatment when their naive pen mates had received oxytocin as compared to a placebo, although this did not differ from the percentage of time “tail low” during the negative treatment with or without oxytocin given to the naive pen mates. These effects of oxytocin on the behavior of the training pigs could be explained if oxytocin has had an effect on the naive pigs which subsequently influenced the training pigs. At present, we can only speculate what this effect was, because we apparently were not able to measure it. Naive and training pigs could not see each other, so perhaps the administration of exogenous oxytocin stimulated the release of endogenous oxytocin (Churchland and Winkielman 2012; Uvnäs-Moberg and Petersson 2005) which subsequently affected the naive pigs’ vocalizations (Seltzer et al. 2010) or pheromone production (Ågren and Lundeberg 2002; Sanchez-Andrade and Kendrick 2009). In the present study, vocalizations were scored, but not per individual pig, and thus, we cannot say whether oxytocin had an effect on the vocalizations of the naive pigs during the positive or negative treatment of the training pigs. However, oxytocin was found to increase the number of low-pitched vocalizations in the emotional contagion test situation which does suggest that vocalizations could underlie the effect of oxytocin on the behavior of the training pigs. These findings could be coincidental as inter-individual effects of oxytocin have—to the best of our knowledge—not been found in human intranasal oxytocin studies, although that could be due to the fact that effects of intranasal oxytocin were only studied in the persons who were also treated with oxytocin. Interestingly, Ågren and co-workers have found effects of oxytocin on the behavior and physiology of rats which had not received oxytocin themselves when exposed to an oxytocin injected cage mate (Ågren and Lundeberg 2002). Therefore, inter-individual effects of oxytocin may merit further research.

## Conclusions

In contrast to two earlier pig studies, the results of this study may provide evidence for emotional contagion in pigs, especially during a negative situation. Surprisingly, no effect of oxytocin was found on the behavior of the pigs which were given an intranasal administration of oxytocin, but some effects of oxytocin were found on the behavior of other pigs which were not treated with oxytocin. This suggests a role for oxytocin in auditory or olfactory communication between pigs as the oxytocin-treated pigs and the other pigs could not see each other.

## Electronic supplementary material

Below is the link to the electronic supplementary material.

Supplementary material 1 (DOCX 18 kb)
