# Supplementary material for: Emotions on the loose: emotional contagion and the role of oxytocin in pigs
Source: Anim Cogn. 2014 Nov 11;18(2):517–32. doi: 10.1007/s10071-014-0820-6 (PMC4320767; doi:10.1007/s10071-014-0820-6)
Supplement: Supplementary file 1 — Supplementary material 1 (DOCX 18 kb) [file 10071_2014_820_MOESM1_ESM.docx]

Online Resource 1

Animal Cognition

# Emotions on the loose: emotional contagion and the role of oxytocin in pigs

# Inonge Reimert^*^, J. Elizabeth Bolhuis, Bas Kemp and T. Bas Rodenburg

^*^corresponding author:

Inonge Reimert

Wageningen University, Department of Animal Sciences, Adaptation Physiology Group, P.O. Box 338, 6700 AH Wageningen, The Netherlands

E-mail: [inonge.reimert@wur.nl](mailto:inonge.reimert@wur.nl)

**Behavioral scoring**

The vocalizations (see Table 2) were scored as events or discrete behaviors by one observer throughout the experiment during the actual trials (i.e. live observations), because it was not possible to score the vocalizations from video as the video cameras could not record sound.

Defecating and urinating were scored after each trial by counting the number of fecal droppings and the presence or absence of urine, respectively, in each compartment. They are therefore also scored as events or discrete behaviors (see Table 2). In addition, they were scored by the same observer who scored the vocalizations. This observer had much experience in scoring different types of vocalizations and in scoring defecations and urinations (see Reimert et al. (2013) Physiol Behav 109:42-50; Reimert et al. (2014) Appl Anim Behav Sci 151:24-35; Reimert et al. (2014) Physiol Behav 129:221-229). She was thus trained to score these behaviors objectively.

The other behaviors in Table 2 were scored from the video recordings made using the Observer software of Noldus Information Technology B.V., Wageningen, The Netherlands. In Observer, an ethogram was made consisting of different behavioral categories (e.g. “tail postures” was such a group (see Table 2 for the other categories)). The behaviors within one category are mutually exclusive and thus cannot occur simultaneously (e.g. a pig has its tail either in a curl, low or wagging posture), but behaviors from different categories can occur simultaneously (e.g. a pig can play and wag its tail at the same time). The ethogram within the software package Observer was set up in such a way that these behaviors were identified as states (which is mentioned in the title of Table 2). For scoring, a video file with the appropriate trial was uploaded to Observer and positioned at the correct time (i.e. the start of the trial). The behavior of the pigs seen, was then scored using the ethogram made (e.g. a pig’s tail is curled: the curl posture is selected, if the tail is later on wagging, the wagging posture is selected, etc.). In this way, the Observer can automatically calculate the duration of each behavior and thereby the percentage of time it occurred. Thereafter, this information was exported to Excel to further analyze the data. For more information regarding Observer, we kindly refer to the following tutorial made by Noldus: <http://www.noldus.com/files/swf/observerxt_tutorial/2013/the_observer_xt_11_tutorial.html>

There were two observers scoring the behaviors of the pigs with Observer. One observer scored the behaviors of the pigs during the positive and negative treatment on test days 23, 24 and 26 and the other on test days 30-33. These two observers were instructed how each behavior looked like (e.g. when a tail is in the wagging posture and when is it not) using photos and movie clips showing the behavior, and they learned to work with Observer from an Observer expert. In addition, they also discussed together when to score one behavior or the other to make sure they scored in a similar way. That the observers scored the behaviors during the treatments in an objective and reliable way may be concluded from the fact that these results are relatively comparable to the results of a study in which we studied the same objectives (i.e. Reimert et al. (2013) Physiol Behav 109:42-50) and in which the behaviors were scored by a different observer.
